# Fabrication and characterization of Schwann cell-derived acellular matrix and chemical-ionic dual crosslinking strategy to produce bioinks for peripheral nerve regeneration

**DOI:** 10.1007/s10856-026-07097-0

**Published:** 2026-06-17

**Authors:** Nasera Rizwana, Poorvi Shivakumar, Vipul Agarwal, Manasa Nune

**Affiliations:** 1 Manipal Institute of Regenerative Medicine, Manipal Academy of Higher Education, Manipal, Karnataka India; 2 Department of Materials Science and Engineering, Monash University, Clayton, VIC Australia

## Abstract

Peripheral nerve injury (PNI) is a common neurological problem that can hamper the quality of life causing sensory and motor dysfunction. While small peripheral nerve defects get repaired by themselves, larger defects require tissue engineering substitutes to bridge the nerve gap. One of the recent technologies and strategies to address the peripheral nerve regeneration is through 3D bioprinting. In this study, we show an effective method to fabricate acellular matrix (ACM) from rat Schwann RSC96 cells and incorporate it into alginate hydrogel. We observed that ACM showed presence of ~400 µg/ml of proteins and specific Schwann cell marker (S100) and myelination markers (PMP22, MPZ) with significant DNA reduction, indicating effective decellularization and minimal immunogenicity. Cytochemical and immunofluorescence staining confirmed presence of extracellular matrix (ECM) proteins, collagen I and fibronectin. We also developed a novel dual crosslinking strategy using glutaraldehyde (pre-print crosslinking) and calcium chloride (post-print crosslinking) to facilitate efficient bioprinting. Further, rat Schwann RSC96 cells were blended with alginate/ACM hydrogel to prepare a functional bioink. Our results demonstrated good cell viability and functionality in the printed structures, highlighting the potential role of rat Schwann RSC96-derived ACM as well as a dual crosslinking strategy for peripheral nerve tissue regeneration.

**Graphical Abstract:**

**Figure:**
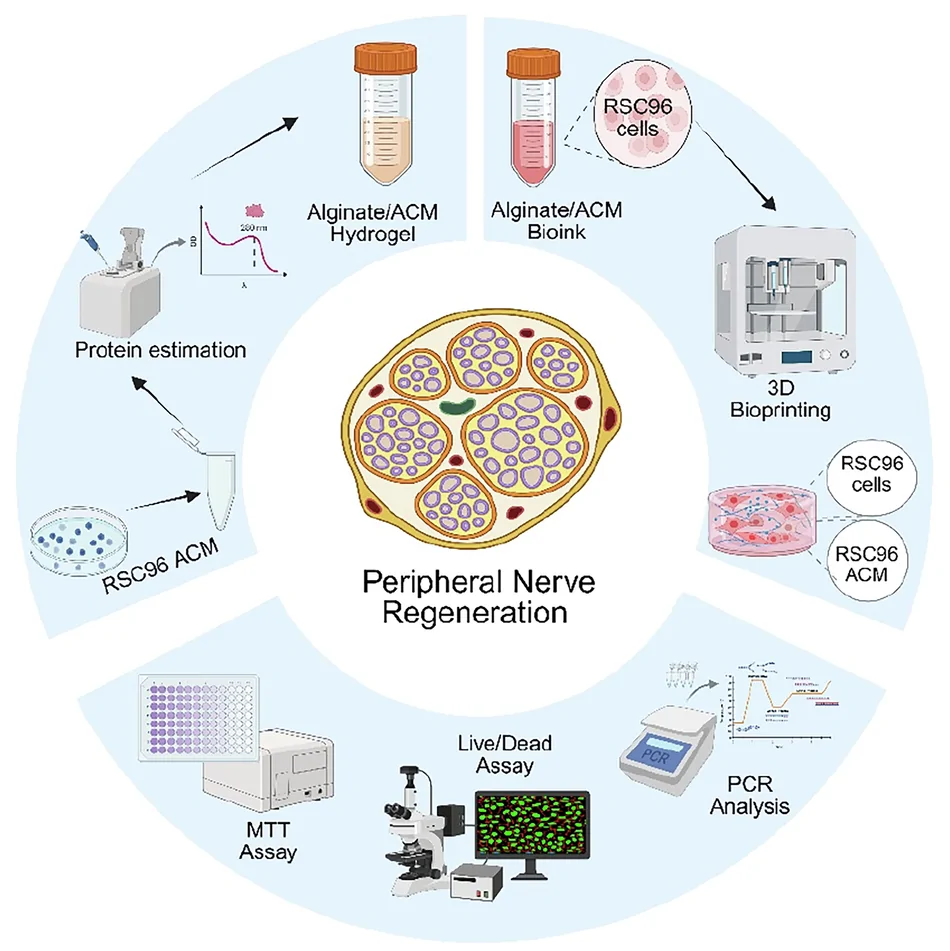
Peripheral nerve injuries can result in significant sensory and motor dysfunction, particularly when the nerve gap is too large for regeneration to take place. To address this challenge, tissue-engineered solutions have been explored, with 3D bioprinting emerging as a promising strategy for creating biomimetic nerve guidance conduits. This study introduces a novel bioink composed of alginate hydrogel integrated with a cell-derived acellular matrix (ACM) obtained from rat Schwann RSC96 cells. ACM provides a biomimetic environment that closely resembles native extracellular matrix, offering enhanced support for cellular functions critical to nerve regeneration. A dual crosslinking strategy using glutaraldehyde and calcium chloride was developed to improve the shape fidelity of the printed constructs. The bioink demonstrated good printability and biocompatibility and supported high cell viability. This approach successfully mimics the natural cellular microenvironment and facilitates Schwann cell functionality, which is crucial for peripheral nerve regeneration. This study presents an efficient method for fabricating functional ACM-based bioinks and highlights the synergistic role of dual crosslinking in achieving 3D bioprinted structures.

## Introduction

Peripheral nerve injury is one of the most common types of traumatic injuries with high prevalence in young population. Currently, gold standard treatment of PNI is the use of autologous nerve grafts [1–6]. However, there are many limitations to autografts like limited donor availability and immune rejection. Hence, it is imperative to develop alternatives to autografts. The application of 3D bioprinting provides a promising replacement to overcome the limitations of autografts. Various synthetic and natural biomaterials are used to fabricate nerve grafts. Synthetic nerve grafts have been known to have low cell affinity and poor hydrophilicity. Therefore, natural biomaterials have been sought for native ECM mimicking characteristics and better cell-scaffold interactions [7, 8].

**Fig. 1:**
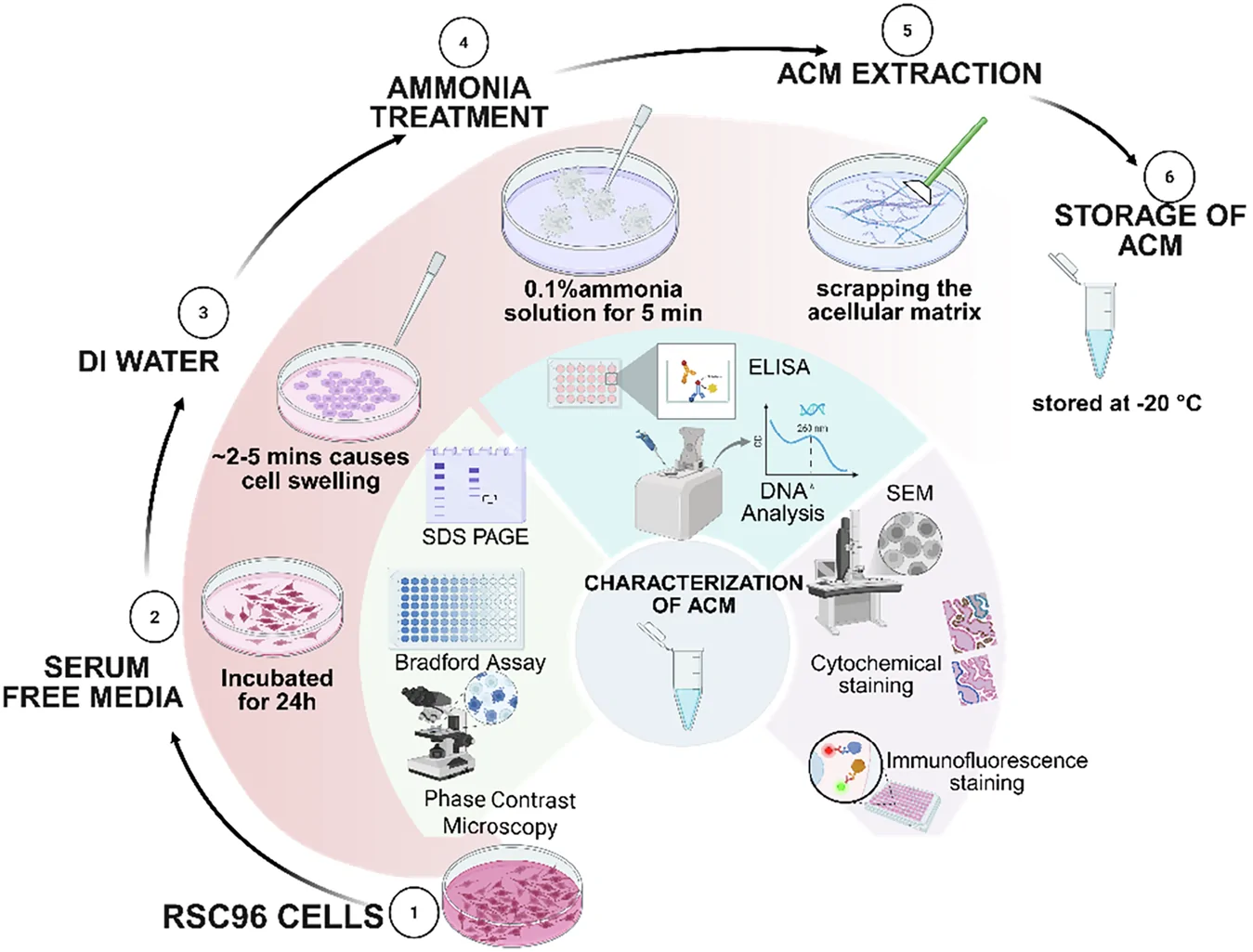
A schematic representing methodology and characterization of acellular matrix (Created using BioRender.com)

ECM is a non-cellular complex network made up of several multidomain macromolecules arranged according to the cell or tissue in which they are found. The mechanical characteristics of the tissues are influenced by the structural stability of the composite formed by the linking of ECM components. Additionally, growth factors and bioactive compounds are also stored in the ECM. ECM plays a crucial role in regulating and shaping the fundamental behaviors and traits of cells including cell proliferation, adhesion, migration, polarity, differentiation, and death. Cells interact with elements of the ECM such as fibronectin, glycosaminoglycans, proteoglycans, thrombospondins, tenascin, vitronectin and collagens. Biochemical signaling factors, various cytokines, and growth factors control the cell interactions with ECM components [9–13]. Decellularized ECM scaffolds have been explored in tissue engineering to repair damaged tissue. The advantages of decellularized ECM include built-in structural and biochemical similarities to the target tissue, biological recognition, and low to no immunogenic response. ECMs can be harvested from two major sources, i.e. tissue-derived and cell-derived. Tissue-derived ECM has various disadvantages such as immune rejection if complete cell removal has not occurred and the composition cannot be tailored according to the tissue of interest [14–16]. Therefore, cell-derived ECM serves as a better alternative for use in regenerative medicine. We refer to these cell-derived decellularized ECM as acellular matrix hereafter. ACM provides various advantages over tissue-derived ECM. Firstly, cultured cells are pathogen free and can be obtained from autologous source or cell lines. Secondly, ACM provides the desired geometry and microenvironment for good cell penetration (Fig. 1). Thirdly, ACM also provides flexibility in combining ECM from various cell types. Additionally, ACM also form a reservoir of various cytokines and growth factors [17–20].

Cell type is an important factor that determines the ECM composition. Cells from various tissues lay down matrices that mimic the natural tissue matrix. Both primary cells and cell lines have been used to obtain cell-derived matrices, which have their own advantages and disadvantages [21]. Although primary cells resemble the native microenvironment closely, obtaining sufficient number of cells has been challenging. Alternatively, cell lines have been used to obtain cell-derived matrices as they are easily cultured and large number of homogeneous cells can be obtained [21]. In peripheral nerve, Schwann cells form the major glial cells. Schwann cell-derived ACM has been reported to aid organization of peripheral nerves by enhancing the adhesion and proliferation of cultured Schwann cells [12, 13, 22–24]. To utilize the properties of ACM, blending it with other bioprintable materials and crosslinking strategies must be employed. A major factor that needs to be considered while incorporating ACM into bioprintable hydrogels is that the characteristics of ACM should not be altered, and ACM should be uniformly blended within the base hydrogel.

Sodium alginate is a linear polysaccharide, alginic acid derivative made up of 1,4-d-mannuronic (M) and -l-guluronic (G) acids. It is one of the most used bioinks due to its various properties. It is a negatively charged biopolymer into which water and other molecules can be encapsulated by capillary action. Due to this, it can also be used to diffuse the media with growth factors, making it an ideal bioink for 3D bioprinting. Sodium alginate most commonly undergoes fast gelation when subjected to calcium ions by forming ionic bridges with the M and G blocks. For bioprinting, bioinks must have good rheological properties like high viscosity and shear thinning. Sodium alginate shows increase in viscosity as the concentration increases. Park et al. [25] demonstrated that 3% sodium alginate solution provided good printability. However, to increase the printability window of sodium alginate, further modifications in crosslinking or incorporation of other polymers are needed. Datta et al. [26], incorporated natural honey along with alginate to improve mechanical and biological properties of the bioink without effecting its rheological properties. One of the major disadvantages of alginate is its fast degradation, which can also be tackled by incorporating other polymers and by designing various crosslinking strategies. Bouhadir et al. [27] reduced the rate of degradation in a controlled manner by using oxidized alginate with sodium periodate. In another study, Boontheekul et al. [28] controlled the degradation rate by partially oxidizing alginate with sodium periodate and incorporating low molecular weight alginate. Relatively fast degradation of neat alginate and lack of maintaining long-term structural integrity proves to be a hindrance in fabrication of bioprinted scaffolds. To address this limitation, we developed a dual chemical-ionic crosslinking strategy by incorporating glutaraldehyde in alginate in order to synthesize the desired ACM bioink and further crosslinking the bioprinted scaffolds with calcium chloride. Glutaraldehyde reacts rapidly with amine groups at around neutral pH and is more efficient than other aldehydes, such as formaldehyde, malonaldehyde in generating thermally and chemically stable crosslinking reactions [29, 30]. Kostenko et al. [31], demonstrated that use of pre-crosslinking of low concentration of alginate with calcium sulphate increased the viscosity of the alginate and rendered it suitable for bioprinting. Studies have also shown that a high concentration of alginate plays a detrimental role in cell viability. Pre-crosslinking of alginate is reported to increase the resolution, printability and shape fidelity of the bioprinted structures [32–34].

The aim of this study was to (i) produce ACM from rat Schwann RSC96 cells and (ii) incorporate it (ACM) into alginate hydrogel to form a bioink. The produced ACM was analyzed using various characterization techniques. We also developed a dual crosslinking strategy by combining chemical (glutaraldehyde) and ionic (calcium chloride) crosslinking methods to produce a printable bioink. The bioink was characterized and the effect of the printed scaffold on rat Schwann RSC96 cell proliferation and gene expression was assessed. To the best of our knowledge, this is the first study in which rat Schwann RSC96 cells-derived ACM was used as a bioink and dual crosslinking strategy in bioprinting of alginate. The produced 3D printed scaffolds were explored for peripheral nerve regeneration in particular for promoting myelination in cultured cells.

## Materials and Methodology

### Materials

Sodium alginate (CAS: 9005-38-3) and phosphate buffer saline (PBS) tablets (P4417) were purchased from Sigma-Aldrich (India). Glutaraldehyde (CAS: 111-30-8) and dimethyl sulfoxide (MB058) were purchased from HiMedia Laboratories Pvt Ltd (India). Calcium chloride (CAS: 10035-04-8) was purchased from SRL Chemicals. Rat Schwann cells (RSC-96) were obtained from American Type Culture Collection (CRL-2765 ATCC, USA). Fetal bovine serum (FBS) (lot no 2440096), antibiotics penicillin-streptomycin (P4333), antibiotic-antimycotic (A5955), and Dulbecco’s Eagle medium (DMEM) (lot no. 2323533) were purchased from Gibco (India). Cytochemical stains (alcian blue (CT-102), Masson’s trichrome, picrosirius red (ST-141), safranin O) were purchased from AMD Labs (India). Coomassie blue and MTT kit (TC191-1G) were purchased from HiMedia Laboratories Pvt Ltd (India). Live/dead kit (lot no. 2015555) was purchased from Invitrogen (USA). Collagen Type 1 Alpha 1 (COL1a1, Cat# PAA35Hu01) was purchased from Cloudclone and Fibronectin rabbit pAb (Cat#A16678) was purchased from ABclonal. Goat anti-rabbit IgG (Cat# 11-4811-86) was purchased from eBioscienece,

### Acellular matrix preparation

Rat Schwann RSC96 cells were cultured in 100 mm dish in culture media consisting of DMEM, 10% FBS, penicillin-streptomycin and antibiotic-antimycotic. Once the cells reached 80–90% confluency, the culture media was discarded, and serum-free media was added and incubated at 37 °C and 5% CO_2_ in a humidified incubator for 24 h. Post incubation, the media were discarded, and DI water was added for 2–5 min to cause swelling of the cells. Once the cells are swollen, the water is discarded, and 0.1% ammonia solution is added for 5 min for decellularization to occur. Post decellularization, gentle copious PBS washes were given to remove the debris. 1 ml of PBS was added to the dish, and ACM was scrapped using a cell scrapper, collected, and stored at −20 °C for further characterizations (Fig. 1).

### Characterization of acellular matrix

#### Phase contrast microscopy

Rat Schwann RSC96 cells were observed at various stages of decellularization under a phase contrast microscope (Nikon Eclipse TE2000-5).

#### Protein estimation by bradford assay

Bradford assay was performed to estimate the protein concentration in the ACM and rat Schwann RSC96 cell lysate. Proteins were extracted from the cell lysate using RIPA buffer. Briefly, 1 mL of RIPA buffer was added to the cell lysate, followed by centrifugation at 10,000 rpm for 10 min at 4 °C to obtain the protein extract. Since ACM represents a collection of secreted proteins, no additional extraction protocol was required for its processing. The protein standard - Bovine Serum Albumin (BSA) was serially diluted with distilled water ranging from 0 to 800 µg/ml. 5 µl of sample was added to 200 µl of Bradford reagent and incubated in dark at room temperature for 5 min. Spectroscopic measurements of absorption were taken at 595 nm using a multiplate reader (HH34000000, Perkin Elmer (Ensight)). The standard curve was plotted, and an *R*^2^ value of 0.9787 was obtained (Fig. S1, (supporting information). The protein concentration of the samples was analyzed using the standard curve.

#### Sodium dodecyl sulfate-polyacrylamide gel electrophoresis (SDS-PAGE)

Prior to running the SDS-PAGE gel, the samples were dissolved in a sample buffer and were heated for 5 min at 95 °C. 30 μl of each sample was loaded into the 8% SDS gel. The gel was run for 30 min at 80 V and at 110 V for another 2 h. The gel was stained overnight with Coomassie Brilliant Blue, and gel images were captured using ChemiDoc XRS+ (BIO-RAD). Cell lysate and ACM sample were prepared as mentioned in section 2.3.2.

#### Enzyme-linked immunosorbent assay (ELISA)

The concentration of myelin proteins like peripheral myelin protein 22 (PMP22), myelin protein zero (MPZ), and S100 were measured using a commercially available rat ELISA kit, according to the manufacturer’s instructions (Krishgen). Cell lysate and ACM sample were prepared as mentioned in section 2.3.2.

#### DNA content analysis

Rat Schwann RSC96 cell lysate and ACM were subjected to DNA quantification. DNA from the cells was isolated using protocol based on phenol/chloroform/isoamyl alcohol method. DNA quantification was performed using Nanodrop One (Thermo Scientific).

#### Scanning electron microscopy (SEM)

Scanning electron microscopy was performed using Zeiss Ultra55. ACM was prepared on round cover slips and fixed with 2.5% glutaraldehyde and subsequently dehydrated using increasing concentrations of ethanol (50%, 60%, 70%, 80%, 90% and 100%) and kept in acetone. The fixed and dehydrated samples were placed on adhesive double-sided carbon tape, which was sputter coated with gold prior to imaging.

#### Cytochemical staining

Rat Schwann RSC96 cells and ACM were subjected to cytochemical staining for detection of major ECM components after fixing them with 2% paraformaldehyde. The presence of collagenous matrix was demonstrated by staining with Masson’s Trichrome kit. Fibrillar collagen was detected by staining with Picro Sirius Red. Proteoglycans and ECM-bound GAGs were detected by staining with Safranin-O and Alcian Blue stain as per the manufacturer's instructions (AMD Labs). The staining was observed under a phase contrast microscope (Nikon ECLIPSE Ts 2R), and images were processed using ImageJ software.

#### Immunocytochemistry

To investigate the proteins, present in the ACM, immunofluorescent staining was performed. Rat Schwann RSC96 cells and ACM were fixed with 2% paraformaldehyde for 20 min at room temperature. The samples were permeabilized with 0.01% Triton X-100 in PBS, blocked with 0.3% BSA. Primary antibodies including polyclonal antibody to Collagen Type 1 Alpha 1 (COL1a1, Cloudclone, Cat# PAA35Hu01) and Fibronectin Rabbit pAb (AB clonal, Cat#A16678) was diluted in required concentration in blocking buffer and was added into the samples and incubated in dark at 4 °C overnight. Post incubation, secondary antibody (Goat anti-rabbit IgG, eBioscienece, Cat# 11-4811-86) was added into the samples and incubated for 4 h at room temperature in dark. After 4 h incubation, the cell nuclei were counterstained with DAPI (1:1000; HiMedia) and incubated for 5 min in dark at room temperature. The samples were washed with PBS and fluorescence images of each sample were taken using a fluorescence microscope (Nikon Eclipse TE2000-U) and recorded by an attached digital camera. The images were analyzed using ImageJ software.

### Alginate/ACM hydrogel preparation

Alginate/ACM hydrogel was prepared by dissolving 3% sodium alginate (Sigma-Aldrich CAS: 9005-38-3) in PBS at 50 °C for 1 h at 600 rpm. After complete dissolution, 2% glutaraldehyde (HiMedia CAS#: 111-30-8) was added and stirred for 15 min at 50 °C at 600 rpm. The homogenized hydrogel was exposed to UV light for 20 min for sterilization. 400 μg/ml of ACM was taken in a 5 ml syringe and 5 ml of alginate hydrogel was taken in another syringe and mixed in to and fro motion until uniformity is obtained. This was labeled as chemical crosslinked alginate/ACM hydrogel.

Alginate hydrogel was prepared by dissolving 3% sodium alginate in PBS at 50 °C for 1 h at 600 rpm. After complete dissolution, 2% glutaraldehyde was added and stirred for 15 min at 50 °C at 600 rpm. This was labeled as a chemical crosslinked alginate hydrogel.

To fabricate lyophilized scaffolds which were used for characterizations, 300 µl of chemical crosslinked alginate and chemical crosslinked alginate/ACM hydrogels were placed in a 48-well plate and subjected to ionic crosslinking using 4% calcium chloride (SRL CAS#10035-04-8) for 2–3 min. These were labeled as dual chemical-ionic crosslinked neat alginate scaffolds and dual chemical-ionic crosslinked alginate/ACM scaffolds. The scaffolds were washed 3 times with PBS and lyophilized.

### Characterization of alginate/ACM hydrogel

#### Cytochemical staining

The dual chemical-ionic crosslinked neat alginate, dual chemical-ionic crosslinked alginate/ACM scaffolds and ACM-coated alginate scaffolds were subjected to cytochemical staining to analyze the presence of ACM within the scaffolds. Chemical-ionic alginate scaffolds were coated with 1 ml of ACM for the preparation of ACM-coated alginate scaffolds. The staining protocols were followed as mentioned in section 2.3.7. The staining was observed under a light microscope (Nikon Eclipse TE2000-U), and images were processed using ImageJ software.

#### Swelling behavior

The in vitro swelling property of the lyophilized scaffolds was studied. Samples were weighed (*w*_i_) and immersed in PBS and incubated at 37 °C. At various time points, the scaffolds were dabbed with tissue to remove excess PBS and weighed (*w*_f_) until saturation. The experiment was performed in triplicate, and swelling ratio was calculated using Eq. (1).

1 $${Swelling}\,{ratio}\,\left( \% \right)=\left(\frac{\,{w}_{f}-\,{w}_{i}}{{w}_{i}}\right)\times 100$$

#### Degradation study

The degradation behavior of the lyophilized scaffolds was assessed by measuring mass loss over a period. The lyophilized scaffolds were weighed (w_i_) and immersed in PBS and incubated at 37 °C. To measure the weight loss, samples were removed from the PBS and dabbed with tissue to remove excess PBS and weighed (*w*_f_) at various time points. The experiment was carried out in triplicate. The degradation rate (%) was calculated using Eq. (2).

2 $${Degradation}\,{ratio}\,\left( \% \right)=\left(\frac{{w}_{i}-{w}_{f}}{{w}_{i}}\right)\times 100$$

#### Rheological behavior of hydrogel

The rheological properties of the chemical crosslinked alginate/ACM hydrogel were evaluated using a rheometer (MCR302 Anton Paar). The dynamic and loss moduli of the hydrogel were measured in oscillatory mode using 25 mm parallel plate at room temperature. 1 ml of hydrogel was used for analysis. Amplitude sweeps and shear thinning behavior were analyzed at a constant angular frequency of 10 rad/s.

### Alginate/ACM bioink preparation and bioprinting

Rat Schwann RSC96 cells were cultured using the same protocol as mentioned in ACM preparation (section 2.2). 1 × 10^4^ cells/ml in 200 µl of media was taken in a 5 ml syringe and homogenously mixed with 3 ml of alginate/ACM hydrogel using gentle to and fro motion to prepare a bioink. The bioink was transferred to the printing cartridge gently without incorporation of air bubbles. Printing cartridge was loaded into the Cellink Inkredible+ bioprinter, and printing was carried out with 27-gauge nozzle at 16 psi pressure. Grid pattern of 10 × 10 mm dimension was printed at a feed rate of 600 mm/min with a line spacing of 2 mm. Post printing, the bioprinted 3D structure was stabilized by subjecting it to 4% calcium chloride solution for 2–3 min. The calcium chloride solution was discarded, and the 3D structure was washed with PBS for 3 times. The bioprinted structure was placed in a humidified incubator at 37 °C and 5% CO_2_ after adding the cell culture media. Cell viability, proliferation and gene expression studies were performed using the cells incorporated within the bioprinted scaffolds.

### Cell viability of bioprinted structure

The cell viability of the bioink was assessed by live/dead assay at days 1 and 5. To prepare the working solutions of calcein-AM and ethidium homodimer, 1 μl of calcein-AM was added to 1 ml of PBS and 4 μl of ethidium homodimer was added to another 1 ml of PBS. 100 μl of calcein-AM working solution was added to the bioprinted scaffold and incubated for 20 min in dark in a humidified incubator at 37 °C and 5% CO_2_. Post incubation, 100 μl of ethidium homodimer working solution was added to the bioprinted scaffold and incubated for 10 mins in dark in humidified incubator at 37 °C and 5% CO_2_. The dyes were aspirated out, and the bioprinted structure was kept in PBS to avoid curling. The 3D bioprinted scaffolds were observed and imaged using fluorescence microscopy (Nikon Eclipse-TE2000-U). The images were analyzed using ImageJ software.

### Cell proliferation of bioprinted structure

Cell proliferation studies of rat Schwann RSC96 cells in the bioprinted alginate/ACM scaffolds were conducted using MTT assay (HiMedia CAS# 298-93-1) as per the manufacturer’s protocol. Rat Schwann RSC96 cells were incorporated into the chemically crosslinked alginate and alginate/ACM hydrogels at a density of 1 × 10^4^ cells/ml to form bioinks. The bioinks were printed into a 48-well plate and ionic crosslinking was done using 4% calcium chloride for 2–3 min. Post crosslinking, scaffolds were washed with PBS three times and were incubated in a humidified incubator at 37 °C and 5% CO_2_. On days 1, 3 and 5, media was discarded, 200 µl of 0.5 mg/ml MTT reagent was added and incubated for 4 h for the formazan crystals to be formed. Post incubation, 100 µl DMSO was added to dissolve the formazan crystals and absorbance was measured at 570 nm using a multiplate reader (HH34000000, Perkin Elmer (Ensight)). MTT protocol was also followed for the control scaffolds (scaffolds without cells), and the absorbance was subtracted from the sample absorbance to plot the graph. The graphs were plotted using GraphPad Prism software.

### qRT-PCR analysis

Total RNA was extracted from bioprinted alginate/ACM scaffolds in triplicate at day point 7 using Trizol method with slight modification. Initially, the scaffolds were subjected to 100 mM of sodium citrate and centrifuged at 15,000 rpm at 4 °C for 15 min. The supernatant was collected, and 1 ml of Trizol was added, and standard isolation protocol was followed [35, 36]. The quality and purity of the extracted RNA were evaluated using spectrophotometric measurements (A260/A280 ratio). The samples within the range ~1.8–2.0 were used for cDNA synthesis and PCR analysis. cDNA was prepared using Gbiosciences cDNA synthesis kit according to manufacturer’s instructions. PMP22 gene was analyzed and GAPDH was used as a housekeeping gene in quantitative RT-PCR (QuantStudio 5) for 45 cycles. The designed primer is listed below in Table 1.

**Table 1:** Primer sequences used for real-time polymerase chain reaction (RT-PCR)

PRIMER	FORWARD SEQUENCE	REVERSE SEQUENCE
GAPDH	GACATGCCGCCTGGAGAAAC	AGCCCAGGATGCCCTTTAGT
PMP22	AATAATCCGCTGCCCGAATCAAG	CTCCGCCTTCAGGGTCAAGTC

The qRT-PCR cycle including denaturation, annealing, and extending phases occurred at 95 °C, 53 °C, and 94 °C for 30, 30, and 5 s, respectively. Quantitative expression of the gene was calculated after normalizing against the endogenous housekeeping gene GAPDH and the data was analyzed using the delta-delta CT method (*n* = 3). The gene expression results were tested with the two-way analysis of variance (ANOVA) using GraphPad Prism.

### Statistical analysis

All the quantitative data of the results are presented as mean ± standard deviation (SD). The results were analyzed using two-tailed Unpaired *t*-test, two-way ANOVA and Tukey’s multiple comparison test and Ordinary one-way ANOVA and Sidak’s multiple comparison test. Statistical analysis was conducted with at least 3 independent samples per experiment. A *p* value < 0.05 was considered statistically significant.

## Results and discussion

### Characterization of rat Schwann RSC96 cell-derived ACM

#### Phase contrast microscopy

**Fig. 2:**
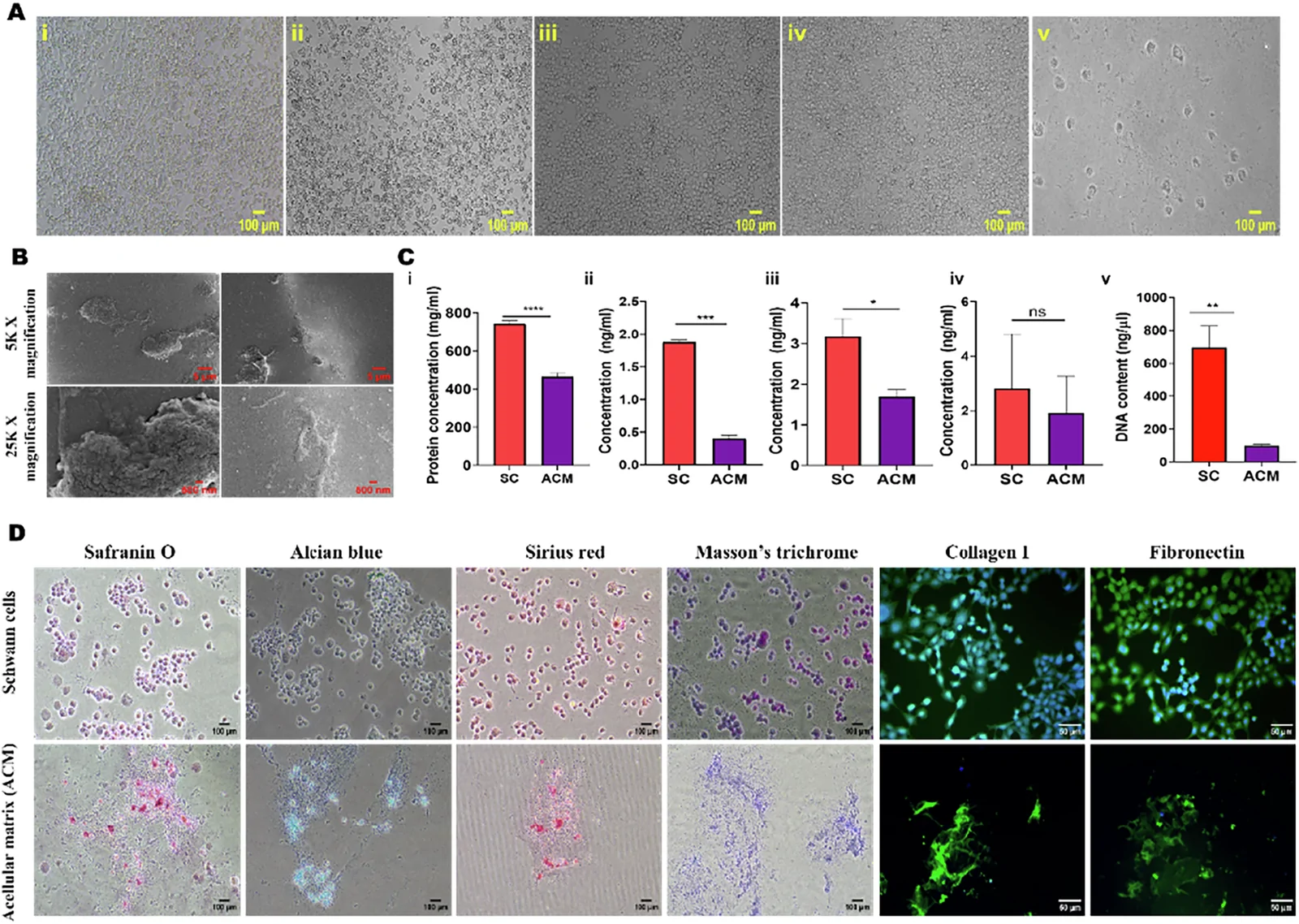
**A** Representative phase contrast microscopy images of various stages of preparation of ACM (i) 90–100% confluent cells (ii) cells 24 h post-serum starvation (iii) swelling of cells after addition of DI water (iv) cells after ammonia treatment and (v) ACM. Scale bar = 100 µm. **B** Representative scanning electron microscopy images of granular bulk structures of ACM. Scale bar = 500 nm and 5 µm. **C** (i) Comparison of protein concentration between rat Schwann RSC96 cell lysate (SC) and acellular matrix (ACM) as measured using the Bradford assay, ELISA plots of (ii) S100 protein, (iii) PMP22, (iv) MPZ and (v) comparison of DNA content between SC and ACM using nanodrop. Statistical analysis was done using the two-tailed unpaired *t*-test with *: *p* < 0.05, **: *p* < 0.01 ***: *p* < 0.001, ****: *p* < 0.0001 ns: not significant. Data are presented as average ± standard deviation (*n* = 3). **D** Representative microscopy images of cytochemical characterization and fluorescence microscopy images of immunofluorescence characterization of rat Schwann RSC96 cells and ACM. Scale bar = 100 µm and 50 µm

Rat Schwann RSC96 cells were cultured for 2 days till 90–100% confluency was achieved and were subjected to decellularization protocol to successfully obtain ACM. Phase contrast microscopy was employed to capture images during each step: confluent cells (Fig. 2A(i)), cells post-serum starvation (Fig. 2A(ii)), swollen cells after DI water treatment (Fig. 2A(iii)), cells after ammonia treatment (Fig. 2A(iv)) and small irregular structures representing ACM (Fig. 2A(v)). A progressive loss of cellular morphology with visible formation of non-cellular matrix-like structures was observed across the stages.

#### Scanning Electron Microscopy

Scanning electron microscopy images of ACM demonstrated the ultrastructure and microdetails of the ACM surface. The deposited matrix showed presence of thick granular bulk structures with visible irregular morphology (Fig. 2B). The absence of cellular features indicated successful decellularization.

#### Protein and DNA quantification

To evaluate the effect of the decellularization method on the protein content of ACM, Bradford analysis was performed. The protein concentration of rat Schwann RSC96 cell lysate and ACM were calculated using the calibration curve (Fig. S1(a), (supporting information)). It was observed that the protein concentration in ACM was ~ 400–450 µg/ml and in rat Schwann RSC96 cell lysate was ~700–800 µg/ml (Fig. 2C(i)). To assess the presence on specific proteins, ELISA was performed. The presence of proteins like S100, PMP22 and MPZ was observed in ACM in reduced concentration when compared to rat Schwann RSC96 cell lysate (Fig. 2C (ii, iii, iv)). The ACM was further subjected to SDS PAGE analysis to confirm the presence of proteins. In the case of ACM, multiple bands were observed at 40 kDa, 52 kDa, 66 kDa, 80 kDa, while in the case of rat Schwann RSC96 cell lysate, bands were visible only below 66 kDa (Fig. S1(b), (supporting information). The presence of cellular DNA post decellularization was quantified using the Nanodrop method (Fig. 2C(v)). The results indicated that the DNA content in rat Schwann RSC96 cells was ~650–700 ng/ml, while that in ACM was below 100 ng/ml.

#### Cytochemical and immunofluorescence characterization

The components of ACM were characterized using cytochemical staining. Rat Schwann RSC96 cells and ACM were fixed with paraformaldehyde and were subjected to Safranin O (glycosaminoglycans (GAGs)), Alcian blue (proteoglycans), Sirius red (fibrillar collagen) and Masson’s trichrome (collagen) staining. Irregular patches of stains were observed in ACM, depicting the presence of deposited matrix (Fig. 2D). To confirm the intactness of basal membrane, immunofluorescence staining was performed. The ECM proteins, like collagen I and fibronectin, were analyzed. The deposited matrix was positively stained for collagen I and fibronectin, and nuclear staining (DAPI) was absent in ACM when compared to rat Schwann RSC96 cells (Fig. 2D).

### Characterization of alginate/ACM hydrogel

#### Cytochemical staining

**Fig. 3:**
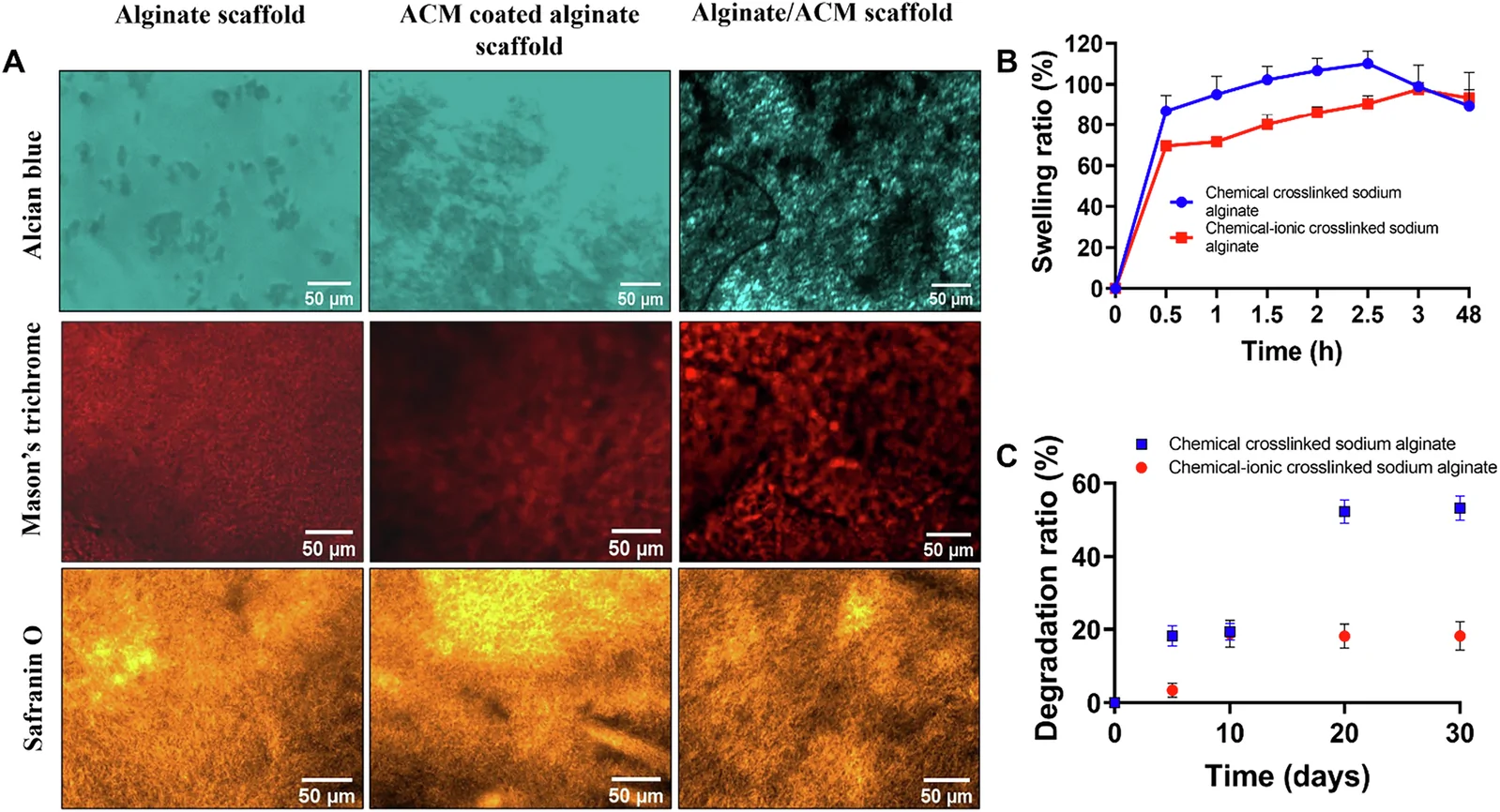
**A** Representative microscopic images of cytochemical characterization comparing alginate scaffold as control, ACM-coated alginate scaffold, and alginate/ACM scaffold with Alcian blue, Masson’s trichrome, and Safranine O. Scale bar = 50 µm. **B** Swelling behavior and (**C**) Degradation analysis of chemical crosslinked sodium alginate and dual chemical-ionic crosslinked sodium alginate scaffolds in PBS

To evaluate the proper blending of ACM in the prepared alginate hydrogel, cytochemical staining was carried out. Alcian blue, Masson’s trichrome, and Safranine O staining were conducted on three samples: alginate scaffolds as a control, ACM coated on alginate scaffolds, and alginate/ACM scaffolds. Based on the visual inspection, we postulate an increase in the intensity of all markers (Alcian blue, Masson’s trichrome, and Safranine O) in alginate/ACM scaffolds compared to the ACM-coated alginate scaffold and the control scaffold, potentially indicating that the ACM was uniformly incorporated within the alginate hydrogel using our technique (Fig. 3A).

#### Swelling and degradation analysis

The swelling and degradation properties for lyophilized chemical crosslinked and dual chemical-ionic crosslinked alginate scaffolds were investigated in PBS. Chemical crosslinked alginate scaffolds exhibited 80–90% swelling in the initial 0.5 h and further increased up to 100% within 2.5 h (Fig. 3B). A gradual reduction in swelling was observed after 3 h (85–90%), which continued until 48 h (80%). In the case of dual chemical-ionic crosslinked alginate scaffold, swelling increased from ~70% at 0.5 h to the maximum of ~90% at 2.5 h and then started to reduce gradually until 48 h reaching ~85%.

In the degradation study, chemical crosslinked alginate scaffolds showed ~20% degradation while dual chemical-ionic crosslinked alginate scaffolds showed ~5% degradation at day 5 (Fig. 3C). By day 20, the hydrogel degradation increased to ~50% for chemical crosslinked alginate scaffolds compared to only ~20% for dual chemical-ionic crosslinked alginate scaffolds. Compared to chemical crosslinked hydrogel, which continued to degrade with time, no further change in dual chemical-ionic crosslinked hydrogel was observed past 20 to 30 days.

#### Rheological characterization and bioprinting

**Fig. 4:**
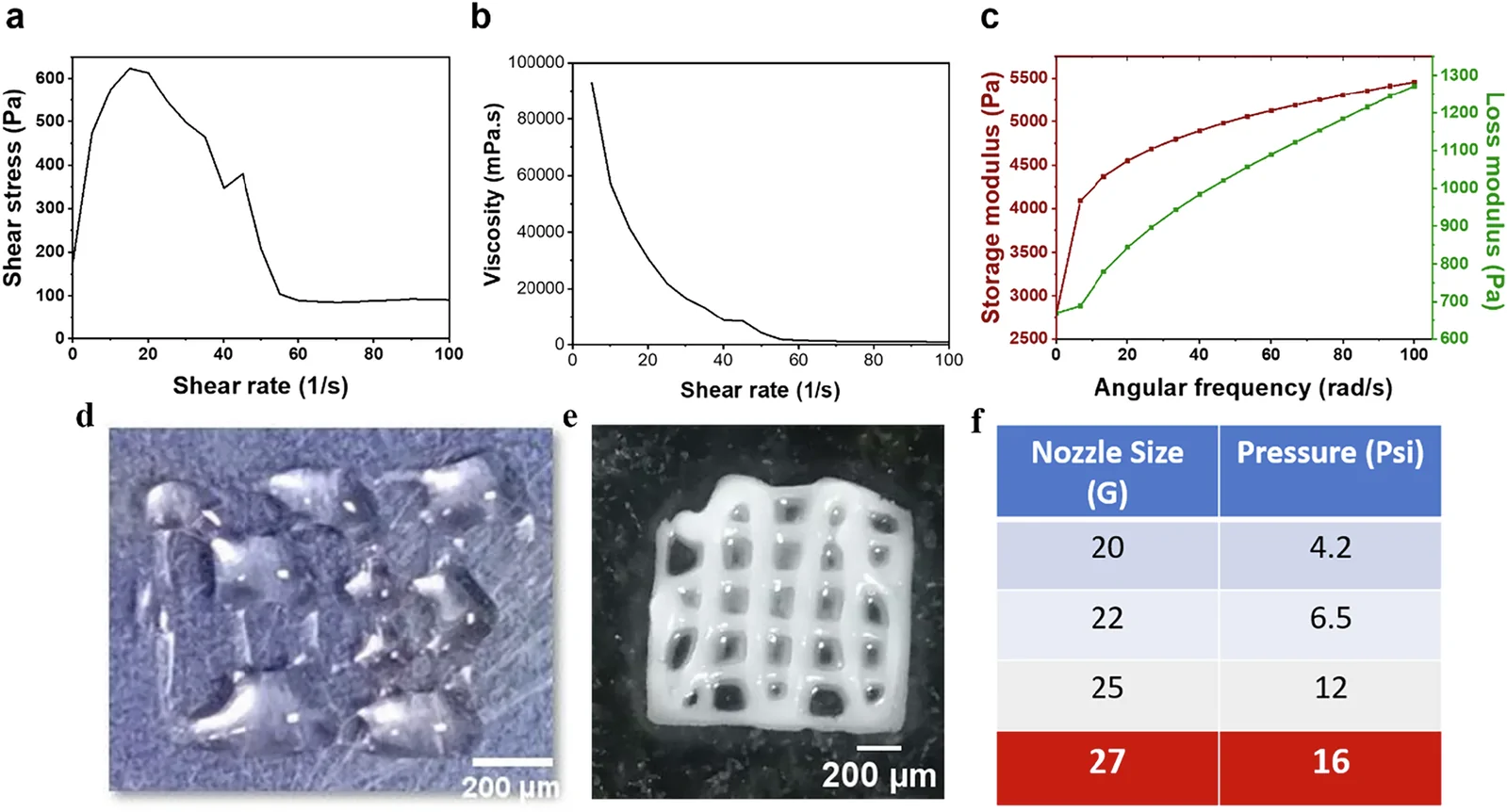
Rheological behavior of chemical crosslinked alginate/ACM hydrogel (**a**) shear stress vs shear rate, (**b**) viscosity vs shear rate at 37 °C, and (**c**) dynamic modulus at varying angular frequency. Representative macroscopic images for evaluating bioprinting (**d**) improper printing with 25-gauge nozzle, and (**e**) 10 × 10 mm grid structure printed with a 27-gauge nozzle with post-print crosslinking using calcium chloride, and (**f**) table representing optimization of various nozzle sizes and pressure at which print was obtained, red highlight indicates the pressure and nozzle size to obtain good printing. Scale bar = 200 µm

Rheological analysis was conducted only on the printable hydrogel formulation. Figure 4(a) illustrates shear stress as a function of the shear rate of alginate/ACM hydrogel. The hydrogel exhibited an initial increase in shear stress upto ~15 s^−1^ and further showing decrease in shear stress. The frequency sweep (Fig. 4(c)) was performed to measure storage modulus (G’) and loss modulus (G”). The storage modulus was found to be greater than the loss modulus when measured against throughout the frequency range at 37 °C.

The chemical crosslinked hydrogel was transferred to the printing cartridge, and a test print of 10 × 10 mm grid pattern was carried out. The grid pattern was printed at a feed rate of 600 mm/min with a line spacing of 2 mm. As seen in Fig. 4(d), a 25-gauge nozzle with 12 psi pressure was able to extrude the hydrogel, but a proper smooth print was not obtained. However, with a 27-gauge nozzle and 16 psi pressure (Fig. 4(e)), a proper printed grid structure with smooth filaments was achieved. The different nozzle sizes and printing pressures used for optimization have been mentioned in Fig. 4(f). Post-printing, ionic crosslinking with calcium chloride was done to obtain shape fidelity. For bioprinting, alginate/ACM hydrogel was prepared by adding 400 μg/ml of ACM to chemically crosslinked alginate using a to-and-fro motion involving two syringes.

### Cell viability, proliferation, and gene expression studies

**Fig. 5:**
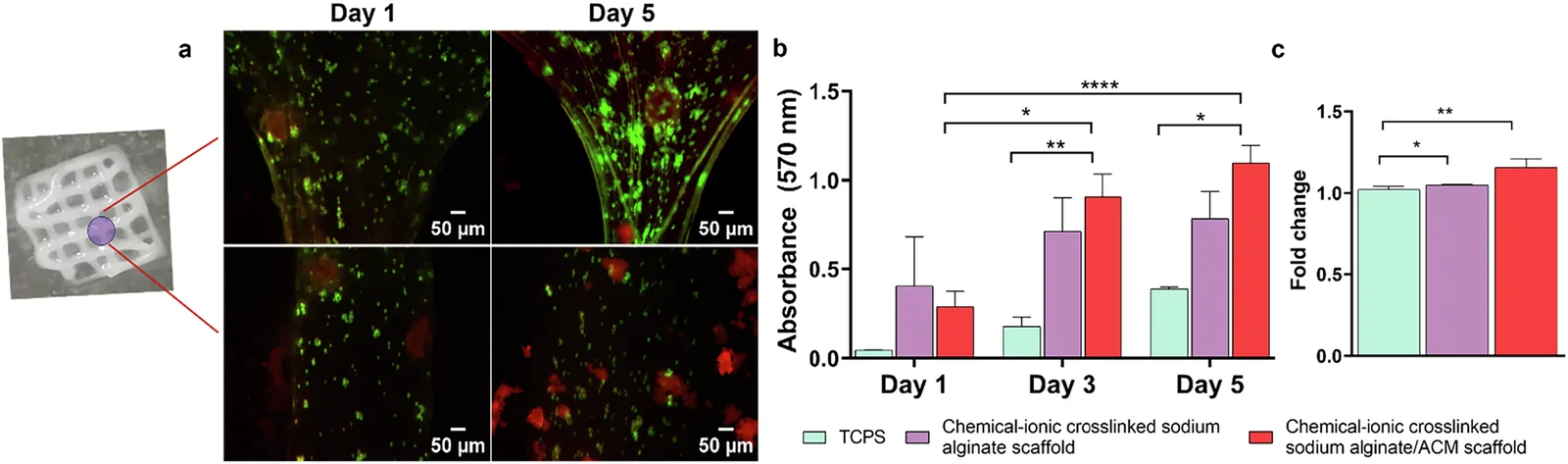
**a** Representative fluorescence images of live/dead assay of bioprinted structure at day 1 and day 5, (**b**) MTT assay evaluating cell proliferation of alginate bioink and alginate/ACM bioink and (**c**) gene expression analysis of PMP22 gene. Statistical analysis was done using two-way ANOVA and Tukey’s multiple comparison test for MTT assay and Ordinary one-way ANOVA and Sidak’s multiple comparison test for gene expression analysis with *p < 0.05, **p < 0.01, and ****p < 0.0001. Data is represented as average ± standard deviation (*n* = 3). Scale bar = 50 µm

Figure 5(a) showed that rat Schwann RSC96 cells remained viable after extrusion-based bioprinting in a live/dead assay. Furthermore, encapsulated rat Schwann RSC96 cells showed good viability after day 5 of culture, indicating that the method used to design the alginate/ACM bioink and bioprinting parameters employed were compatible. To evaluate the effect of glutaraldehyde crosslinking on cell proliferation, an initial MTT assay using rat Schwann RSC96 cells on sodium alginate, chemical crosslinked sodium alginate and chemical-ionic crosslinked sodium alginate scaffolds was performed (Supporting Information, Fig. S3). It was observed that cell proliferation increased progressively from day 1 to day 5 across all scaffolds (sodium alginate, chemical crosslinked sodium alginate, and chemical-ionic crosslinked sodium alginate). The concentration of glutaraldehyde within the scaffolds did not adversely affect cell proliferation under the experimental conditions, confirming its cytocompatibility.

Following the validation that glutaraldehyde was cytocompatible, a second MTT assay was performed to assess the proliferation of cells within the bioink, using rat Schwann RSC96 cells (Fig. 5(b)). The assay was conducted at three different time points (day 1, 3, and 5) with 10,000 cells incorporated into the hydrogels to prepare bioink, and absorbance was measured at 570 nm in a multiplate reader. Chemical-ionic crosslinked alginate/ACM bioink was compared with chemical-ionic crosslinked alginate bioink, and TCPS control. It was observed that there was a significant increase in cell proliferation in alginate/ACM bioink from day 1 to day 5. Additionally, it was also observed that cell proliferation was significantly higher in alginate/ACM bioink when compared to alginate bioink at all time points. The effect of bioprinting and ACM on myelination was analyzed by qRT-PCR analysis for expression of PMP22 gene. It was observed that PMP22 gene expression was significantly upregulated in alginate/ACM bioink when compared to alginate bioink and TCPS control (Fig. 5(c)).

## Discussion

After attaining the desired confluency (Fig. 2A(i)), cells were subjected to serum starvation for 30 min to enhance collagenous proteins [37, 38] as the exogenous matrix metalloproteases present in the serum cause degradation of ECM and disrupts ECM’s remodeling rate (Fig. 2A(ii)) [38–40]. The change in the shape of the cells from spindle shape to round was observed as seen in Fig. 2A(iii) due to the inward movement of water via osmosis, which leads to increase in the absorption of detergent into the cell membrane and causes osmotic lysis [41]. Finally, cells were treated with 0.1% ammonia (Fig. 2A(iv)) to achieve complete decellularization by dehydrating cells and detaching the cellular materials from the ECM [41]. When observed under phase contrast microscopy, small areas of irregularly shaped structures were observed, indicating presence of the deposited matrix (Fig. 2A(v)). The obtained ACM yield of ~400–450 µg/ml suggested that, though mild treatment was given to cells using the ammonia method for decellularization, a significant amount of reduction in the concentration of proteins in ACM was observed, which was not desired but acceptable. This reduction was anticipated due to the removal of cellular proteins. The basal membrane plays an important role in cell anchorage and cell signaling. Therefore, the proteins present in the basal membrane should be well preserved post decellularization also. This was confirmed by the cytochemical staining in which a heterogenous distribution of dense and loosely packed structures was observed in ACM, indicating that the ammonia method used for decellularization was mild enough to retain the ECM proteins. The DNA content in ACM was also significantly reduced post decellularization which is in agreement with previous studies [42].

Chemical crosslinked alginate scaffolds and dual chemical-ionic crosslinked alginate scaffolds were prepared and lyophilized. The scaffolds were analyzed for their swelling and degradation properties. The good swelling ability of the scaffolds is known to create a moist environment for cell growth and enhance diffusion of nutrients and oxygen [43–45]. It is well accepted that higher crosslinking reduces the swelling ability of a hydrogel. Therefore, the obtained results are as expected, that dual chemical-ionic crosslinked hydrogels swell considerably lower than chemical crosslinked hydrogels. Early degradation (within 30 days) was observed in chemical crosslinked alginate scaffolds. The presence of free alginate groups in chemically crosslinked alginate scaffolds may be the reason for early degradation. When dual chemical-ionic crosslinking was done with calcium chloride, the free alginates were converted into calcium alginate and covalent bonds were formed. This formation of covalent bonds could probably explain the significant reduction in the degradation of dual chemical-ionic crosslinked alginate scaffolds.

The approach used to create a printable hydrogel involved employing dual chemical-ionic crosslinking steps. Initially, glutaraldehyde was added to sodium alginate to the extent that the viscosity of the hydrogel was such that it could be extruded from the bioprinter nozzle. Alginate and glutaraldehyde undergo acid-catalyzed reaction to form an acetal-linked hydrogel network. When glutaraldehyde is used as a crosslinker along with other crosslinkers, it behaves as a bifunctional chemical crosslinker for alginate. It forms an acetal link with the hydroxyl functional groups present in the alginate [29, 46]. Calcium chloride is a common salt used for crosslinking alginate hydrogels. Calcium ions replace the sodium ions from sodium alginate to form calcium alginate and release sodium chloride as a byproduct [47].

The flow behavior of the chemical crosslinked alginate/ACM hydrogel at room temperature was evaluated by measuring the rheological properties. When the hydrogel is subjected to bioprinting, it is exposed to shear stress. Therefore, analyzing the rheological behavior of hydrogel during extrusion process is necessary. This behavior is similar to that of non-Newtonian fluids [48]. Non-Newtonian fluids can be either shear-thinning or shear-thickening, which was analyzed by evaluating viscosity as a function of increasing shear rate. When the viscosity of the chemical crosslinked alginate/ACM hydrogel was measured against shear rate, it was observed that the viscosity decreased with increasing shear rate, confirming the shear thinning behavior (Fig. 4(b)). The shear thinning property helps a hydrogel to be easily extruded out of the nozzle during bioprinting. The storage modulus was found to be greater than the loss modulus, indicating that the hydrogel has a stable quasi-solid-like behavior [49].

To evaluate whether the components of bioink, crosslinking strategy and the printing parameters may affect the cell viability within the bioink, live/dead assay for the alginate/ACM bioprinted structure was performed on days 1 and 5. Good cell viability was observed on both day 1 and 5. However, it was also observed that the bioprinted structure began to disintegrate by day 5, and the hydrogel chunks took up the red stains. To confirm that the hydrogel took up the ethidium homodimer (red) stain, bioprinted scaffolds without cells were also subjected to live/dead staining. It was observed, as seen in Figure S2 (supporting information), that the bioprinted scaffold showed predominantly red fluorescence indicative of passive absorption of ethidium homodimer dye by the scaffold. Cell proliferation was analyzed using MTT assay to evaluate the effect of crosslinking within the scaffolds. The concentration used for chemical crosslinking of sodium alginate in our present work did not adversely affect cell proliferation. Glutaraldehyde is one of the most effective protein crosslinking agents. Although it is known for its potential toxicity, its effects are largely dose-dependent [30, 50, 51]. There was an increase in cell proliferation in alginate/ACM scaffolds, which can be attributed to the bioactive cues and microenvironment provided by the ACM, as suggested in previous literature [52]. Taken together the results of the live/dead assay and MTT assay, alginate/ACM bioink exhibited satisfactory biocompatibility. Further, gene expression analysis was done. PMP22 is a pro-myelinating gene that is expressed by Schwann cells. It plays an important role in myelin sheath formation. In our previous works also, we have observed that ACM has positively influenced the expression of PMP22 gene [13, 24].

## Conclusion

We have developed a simple and efficient method of producing cell-derived ACM from rat Schwann RSC96 cells. The ammonia treatment used for decellularization preserved a significant number of proteins while eliminating the nuclear content, thereby reducing immunogenicity. We also devised a technique to effectively blend ACM with alginate hydrogel, which was confirmed using cytochemical analysis. The alginate/ACM hydrogel was subjected to bioprinting where dual chemical-ionic crosslinking was employed to improve printability and shape fidelity. We incorporated glutaraldehyde as a chemical crosslinker within the alginate/ACM hydrogel to obtain a printable hydrogel, and post-bioprinting, the scaffold was subjected to ionic crosslinking with calcium chloride to maintain structural stability. We also demonstrated that when rat Schwann RSC96 cells were incorporated into alginate/ACM hydrogel to form bioink, the cell viability and cell proliferation were maintained, which depicted that the crosslinking strategy and bioprinting parameters did not negatively impact the cells. Additionally, we also studied the PMP22 gene expression, which is a promyelinating factor expressed by Schwann cells that regulates myelination. It was observed that there was significant upregulation of PMP22 gene when compared to the control bioink. Overall, this study helped in understanding rat Schwann RSC96 cell-derived ACM and its effect as a bioink on promyelinating gene expression. One of the limitations that we encountered during this study was the maintenance of the shape of the bioprinted scaffold over a longer period. Other limitations faced during this study were that the bioink was unable to print various standing structures. In future studies, we would like to focus on improving these limitations by incorporating viscosity-enhancing polymers. The outcome of this work is expected to further promote further research in rat Schwann RSC96 cell-derived ACM and its exploration in 3D bioprinting for nerve and neuronal regeneration applications.

## Supplementary information

Supplementary information
