# Supplementary material for: Fabrication and characterization of Schwann cell-derived acellular matrix and chemical-ionic dual crosslinking strategy to produce bioinks for peripheral nerve regeneration
Source: J Mater Sci Mater Med. 2026 Jun 17;37(1):109. doi: 10.1007/s10856-026-07097-0 (PMC13506550; doi:10.1007/s10856-026-07097-0)
Supplement: Supplementary file 1 [file 10856_2026_7097_MOESM1_ESM.docx]

**Fabrication and Characterization of Schwann Cell-Derived Acellular Matrix and Chemical-Ionic Dual Crosslinking strategy to Produce Bioinks for Peripheral Nerve Regeneration**

Nasera Rizwana,^a^ Poorvi Shivakumar,^a^ Vipul Agarwal,*^b^ Manasa Nune*^a^

^a^ *Manipal Institute of Regenerative Medicine, Bengaluru, Manipal Academy of Higher Education, Manipal 576104, Karnataka, India*

*^b^ Department of Materials Science and Engineering, Monash University, Clayton, VIC, 3800 Australia*

*Corresponding authors: [agarwalvipul84@gmail.com](mailto:agarwalvipul84@gmail.com); [manasa.nune@manipal.edu](mailto:manasa.nune@manipal.edu)

Figure S1: (a) Protein standard curve using Bradford assay, and (b) SDS Page analysis of rat Schwann RSC96 cell lysate (SC) and acellular matrix (ACM), PL represents the molecular weight marker of 10-250 kDa molecular weight range.

Figure S2: Representative fluorescence microscopy images of the live/dead assay of the printed scaffold without cells at day points 1 and 5. Scale bar = 50 µm

Figure S3: MTT assay evaluating cell proliferation of sodium alginate, chemical crosslinked sodium alginate and chemical-ionic crosslinked sodium alginate scaffolds. Statistical analysis was done using two-way ANOVA and Tukey’s multiple comparison test with ** p<0.01, and *** p<0.001. Data is represented as average ± standard deviation (n=3).
